# Effective and selective adsorption of methyl *tert*-butyl ether on ZSM-5 zeolite: a comparative study

**DOI:** 10.3389/fchem.2024.1450233

**Published:** 2024-08-16

**Authors:** Tingyu Hua, Shanshan Li, Jingli Hu, Wei Yan

**Affiliations:** Key Laboratory of Solid Waste Recycling and Resource Recovery, Department of Environmental Science and Engineering, Xi’an Jiaotong University, Xi’an, China

**Keywords:** MTBE, ZSM-5 zeolite, adsorption, fixed-bed column, co-contamination

## Abstract

The extensive use of methyl *tert*-butyl ether (MTBE) as a gasoline additive has caused serious environmental problems that need to be addressed urgently. The feasibility of remediation of MTBE-contaminated groundwater by ZSM-5 zeolite with SiO_2_/Al_2_O_3_ ratio of 50/130/360 was explored. The SiO_2_/Al_2_O_3_ ratio had a great influence on the physicochemical properties and structure, as well as the adsorption and mass transfer of MTBE on ZSM-5. The adsorption of MTBE on zeolites with SiO_2_/Al_2_O_3_ ratios of 50 and 130/360 followed the Langmuir and Freundlich models, respectively, and was controlled by different mass transfer processes. The morphology and adsorption capacity of ZSM-5 (50) and ZSM-5 (130) differed significantly, while the differences between ZSM5-(130) and ZSM-5 (360) were less pronounced. ZSM-5 (360) had higher adsorption capacity and adsorption efficiency for MTBE, and the larger BET surface area, pore volume and stronger hydrophobicity were the key factors to promote MTBE adsorption. Compared to activated carbon (AC), ZSM-5 (360) was more effective for MTBE removal at low concentrations (≤200 mg·L^−1^) and had the advantage of selective adsorption of MTBE with the addition of BTEX. In column adsorption, decreasing the concentration had opposite effects on MTBE removal by ZSM-5 and AC. At 5–10 mg·L^−1^, ZSM-5 (360) column reduced effluent concentration and improved bed utilization and removal efficiency.

## 1 Introduction

Methyl *tert*-butyl ether (MTBE) has been widely used as a gasoline additive since the 1970s to increase octane and improve combustion. MTBE is released to the atmosphere or groundwater from a variety of sources, including industrial discharges, storage tank or pipeline leaks, and automobile or gas station spills (Zhang et al., 2018). MTBE is relatively stable and recalcitrant in the environment due to its high water solubility, low Henry’s law constant, and small partition coefficient (Dehghani Kiadehi et al., 2017). Despite being banned in some countries, it is still the second most common volatile organic compound in shallow groundwater (Levchuk et al., 2014). The contamination of drinking water with MTBE has attracted considerable public concerns due to its unpleasant odor, genotoxicity, and harmful effects on the respiratory and nervous systems (Dibaji et al., 2023; Gulack et al., 2024). Since MTBE poses a serious threat to human health, effective technologies are in an urgent demand to remove MTBE from contaminated environments.

There are a variety of treatment technologies available for the remediation of MTBE contamination. Air stripping and chemical oxidation are not the best choice due to their high cost and generation of toxic secondary pollutants (Rodeghero et al., 2017). Physical adsorption has been shown to be one of the most effective methods for rapid reduction of MTBE in the aqueous phase (Levchuk et al., 2014). Activated carbon (AC), clay minerals, resins and zeolites have been widely used as adsorbents for MTBE removal (Table 1). Among them, zeolites are stable under elevated temperature and acidic conditions, and can be easily regenerated by heat treatment (Anderson Michael, 2000). Moreover, MTBE is often accompanied by organic pollutants (benzene or toluene) in gasoline spills (Aivalioti et al., 2012). To improve the selective adsorption performance of MTBE, synthetic zeolites were proposed to remove MTBE from co-contamination. Zeolite Socony Mobil-5 (ZSM-5, MFI-type framework topology) is a microporous aluminosilicate mineral with good mechanical and hydrothermal stability that has received much attention (Shu et al., 2022). The framework is built from SiO_4_ and AlO_4_ tetrahedral units with two channel systems: sinusoidal 10-membered rings (10-MR) channels interconnected with 10-MR straight channels (Rodeghero et al., 2017). Batch adsorption from low (μg·L^−1^) to high (mg·L^−1^) concentrations confirmed that ZSM-5 had a stronger adsorption capacity compared to other zeolites (Abu-Lail et al., 2010). Zhang et al. (2018) showed that ZSM-5 could effectively adsorb MTBE with little desorption at an initial MTBE concentration of 300 mg·L^−1^. Regeneration studies showed that the adsorption capacity of ZSM-5 remained satisfactory (>85%) after up to four regeneration cycles at 80°C, 150°C, and 300°C (Zhang et al., 2019). Vignola et al. (2008) constructed a permeable reactive barrier (PRB) using ZSM-5 and mordenite near a coastal refinery to remediate groundwater contaminated with MTBE and hydrocarbons, and maintained MTBE concentrations in effluent between 0.4 and 4 mg·L^−1^ for 6 months.

**TABLE 1:** Comparison of adsorption capacity of various materials for MTBE.

Adsorbents	Adsorption capacity/mg·g^−1^	Reference
ZSM-5 (469)	53.55	Zhang et al. (2018)
ZSM-5 (280)	0.67	Abu-Lail et al. (2010)
Beta (35)	25.06	
Mordenite (50)	2.76	
HISIV 3000 (800)	6.78	
GAC	204.10	Chen et al. (2010)
Silicalite-1/diatomite	48.40	Lu et al. (2008)
Carbonaceous resin (Ambersorb 572)	4.97	Hung and Lin (2006)
HDTMA-modified clinoptilolite	91.60	Ghadiri et al. (2010)
ZSM-5 (280)	95.00	Rodeghero et al. (2017)
Nano-perfluorooctyl alumina	23.44	Mirzaei et al. (2013)

Although it has been shown that ZSM-5 can effectively remove MTBE from water, studies on the characterization and mechanisms of MTBE adsorption on ZSM-5 with different SiO_2_/Al_2_O_3_ ratios are limited. Few studies have quantitatively compared the adsorption of MTBE on ZSM-5 and AC in fixed-bed systems under different conditions. In this paper, the effect of SiO_2_/Al_2_O_3_ ratio on MTBE adsorption was analyzed in terms of mass transfer, physicochemical properties and skeleton structure. Moreover, the effectiveness of ZSM-5 and AC in removing MTBE under single and mixed contamination was compared. Furthermore, the influence of concentration on the dynamic adsorption of MTBE on ZSM-5 and AC was investigated, and the advantages and application conditions were explored through model fitting.

## 2 Materials and methods

### 2.1 Chemicals and materials

MTBE (purity, 99%) and other chemicals were purchased from Sinopharm Group Chemical Reagent Co., Ltd. (Shanghai, China). ZSM-5 was purchased from Nankai University Catalyst Plant. Coconut shell-based activated carbon (YK-AC) was provided by Shanghai Activated Carbon Co., Ltd.

### 2.2 Characterization

Micromorphology was observed using the Gemini SEM 500 field emission scanning electron microscope (Carl Zeiss Management Co., Ltd., China). Specific surface area and pore volume were measured with a Builder SSA–4300 (China) aperture and specific surface area analyzer using the nitrogen adsorption-desorption method. Surface functional groups were recorded using a TENSOR 37 FTIR spectrometer (Bruker, Billerica, MA, United States) in the range of 4,000–2,200 cm^−1^ with a resolution of 4 cm^−1^.

### 2.3 Batch adsorption experiment

To study the adsorption kinetics of MTBE on ZSM-5, 0.25 g of adsorbent was added to 50 mL of MTBE solution. The headspace flasks were shaken in a shaker (ZWY-2102C) at 25°C, 150 rpm, and samples were taken at different time intervals (0, 30, 60, 120, 240, 360, and 1,440 min). Diluted were made to keep the measurements within the linear range of the standard curve. The amount of MTBE adsorbed at time t can be calculated from Equation 1 as follows:

$$q_{t}=\frac{\left(C_{0}‐C_{t}\right)V}{m}$$ (1)

where, C_0_ and C_t_ are the concentration of MTBE at initial and any time t, respectively (mg·L^−1^). V is the volume of the solution (L) and m is the mass of adsorbent (g).

Equilibrium adsorption experiments were performed in 100 mL headspace flasks using a fixed adsorbent/liquid ratio (0.25 g adsorbent/50 mL solution) with different initial concentrations of MTBE solutions (100, 500, 800, 1,000, 1,200 and 1,500 mg·L^−1^). The adsorption capacity at equilibrium is calculated using Equation 2:

$$q_{e}=\frac{\left(C_{0}‐C_{e}\right)V}{m}$$ (2)

where, C_0_ and C_e_ are the concentration of MTBE at initial and equilibrium phases, respectively (mg·L^−1^). V is the volume of solution (L) and m is the mass of adsorbent (g).

The experimental data need to be considered in segments (Equation 3) due to different mass transfer limiting factors in the early and late stages of adsorption. The fitted equation is as follows:

$$q_{t}=K_{i}t^{0.5}+c$$ (3)

where, q_t_ is the amount of MTBE adsorbed at time t (mg·L^−^), K_i_ is the intraparticle diffusion constant (mg·g^−1^·min^−1^), and c is the constant related to the boundary thickness.

### 2.4 Fixed-bed column tests

Fixed-bed column tests were performed in a glass column (1 cm inner diameter, 10 cm height) at room temperature. The synthetic solution containing MTBE was controlled by a BT100−2 J peristaltic pump (Langer, China). The adsorption experiments were carried out in up-flow mode with a flow rate of 50 mL·h^−1^ and influent MTBE concentrations of 5/10/30 mg·L^−1^. The effluents were collected at set intervals and the MTBE concentration was measured by GC-MS. Four mathematical models were used to fit the breakthrough curves (see Supplementary Material).

### 2.5 Analytical methods

The concentration of MTBE was determined by headspace Gas Chromatography-Mass Spectrometer (GC-MS) (Li et al., 2019). Briefly, 1 mL of aqueous sample was added into a 2 mL headspace bottle, and then 50 μL headspace sample was injected into the injection port using a 50 μL microinjector. MTBE concentration was measured using a Trace GC Ultra (Thermo/Finnigan, Milan, Italy) gas chromatograph equipped with an HP-5MS capillary column (30 m length, 0.25 mm ID, 0.25 µm film; Agilent, Santa Clara, CA, United States) and a Trace ISQ (Thermo/Finnigan, Austin, TX, United States) mass spectrometric detector. The injection port was set in splitless mode, and the temperatures of injector and ion source were set at 200°C and 230°C, respectively. The oven temperature was initially set at 40°C and then gradually increased to 70°C at a rate of 10°C·min^−1^. The flow rate of carrier gas (Helium 5.0) was 1.0 mL·min^-1^, and the mass spectrometry was operated in electron impact mode at 70 eV in selected ion monitoring (SIM) mode at 73 m/z for MTBE.

### 2.6 Theoretical calculations

All calculations were carried out in the framework of density-functional theory with the projector augmented plane-wave method and implemented in the Vienna *ab initio* simulation package (Kresse and Joubert, 1999). The generalized gradient approximation proposed by Perdew, Burke, and Ernzerh was selected for the exchange-correlation potential (Perdew et al., 1996). The cut-off energy of the plane wave was set to 450 eV. The energy criterion was set to 10–5 eV in the iterative solution of the Kohn-Sham equation. A vacuum layer of 15 Å was added perpendicular to the sheet to avoid artificial interactions between periodic images. The Brillouin zone integration was performed using a 2 × 2 × 1 k-mesh. All structures were relaxed until the residual force on the atoms decreased to less than 0.03 eV/Å. The adsorption energy (E_ads_) can be calculated according to Equation 4:

$$E_{\text{ads}}=E_{A+B}‐\left(E_{A}+E_{B}\right)$$ (4)

where E_ads_ is the energy of MTBE adsorbed on the substrate, E_A_ and E_B_ are the energy of MTBE and substrate, respectively.

## 3 Results and discussion

### 3.1 Physicochemical property analysis

**FIGURE 1:**
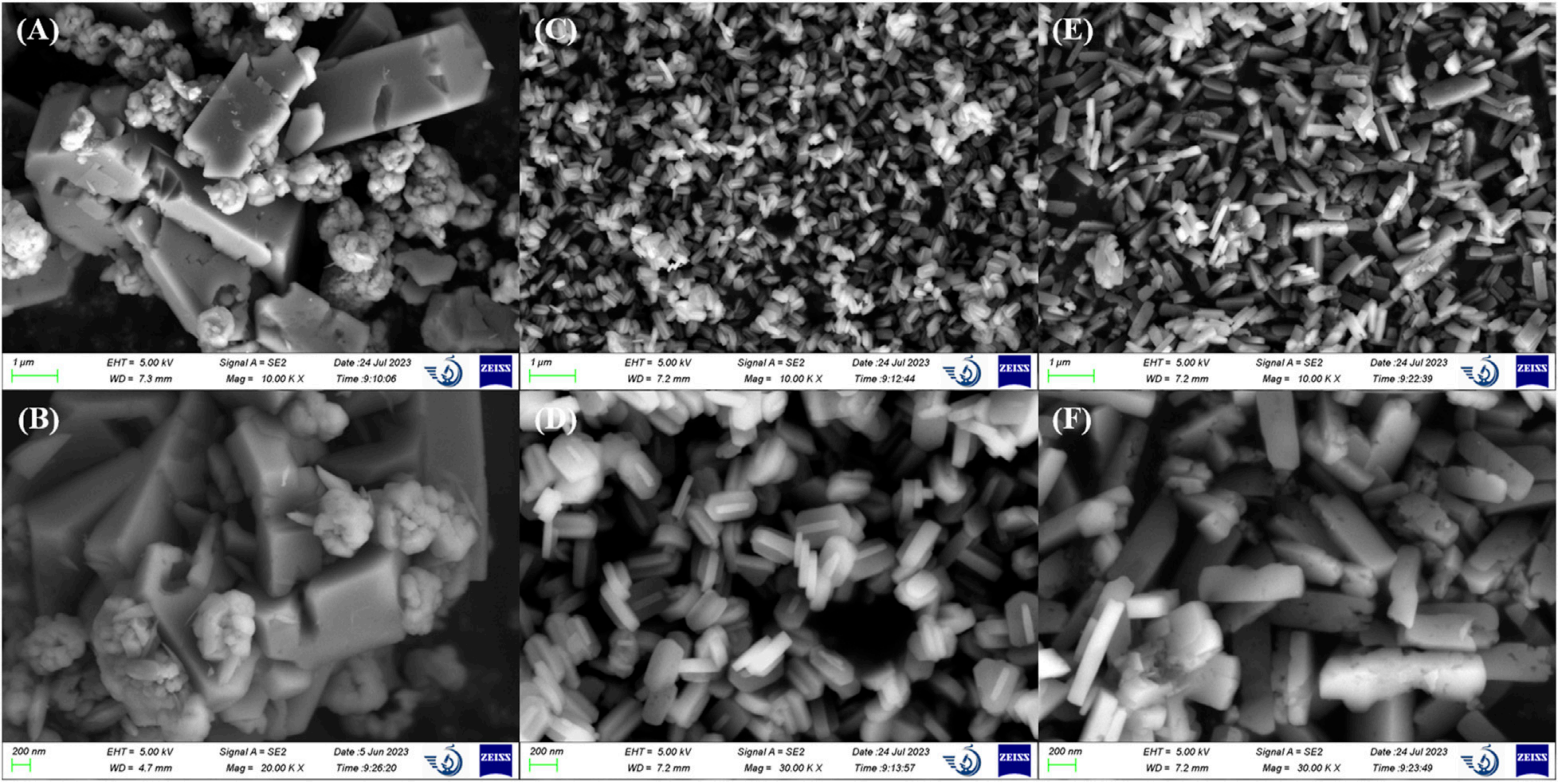
Scanning electron microscopy images of **(A,B)** ZSM-5 (50), **(C,D)** ZSM-5 (130), and **(E,F)** ZSM-5 (360).

ZSM-5 with different SiO_2_/Al_2_O_3_ ratios (50/130/360) were characterized to investigate the differences in structure and chemical properties. As shown in Figure 1, ZSM-5 crystals were nano-sized, and in ZSM-5 (50), a number of small particles were agglomerated on the surface of elongated prisms large than 1 μm. The microstructures of ZSM-5 (130) and ZSM-5 (360) crystals did not differ significantly, and both were stacked with flat hexagonal prisms of uniform size, which proved the excellent crystallinity of zeolite. Among them, ZSM-5 (130) was closer to an elliptical sheet with a length of about 200 nm, and ZSM-5 (360) was more than 400 nm.

The pore size of ZSM-5 is known to be 5.1 Å × 5.5 Å and 5.4 Å × 5.6 Å, and the physical properties of the three ZSM-5 were further compared. As shown in Table 2, the BET surface area and pore volume of ZSM-5 (130) and ZSM-5 (360) were similar, while ZSM-5 (50) was significantly smaller.

**TABLE 2:** Physical property parameters of different ZSM-5.

	BET surface area/m^2^·g^−1^	Pore volume/cm^3^·g^-1^	Pore size (Å)
ZSM-5 (50)	64.88	0.0461	5.1 × 5.5 and 5.4 × 5.6
ZSM-5 (130)	326.06	0.1377	
ZSM-5 (360)	352.10	0.1383	

**FIGURE 2:**
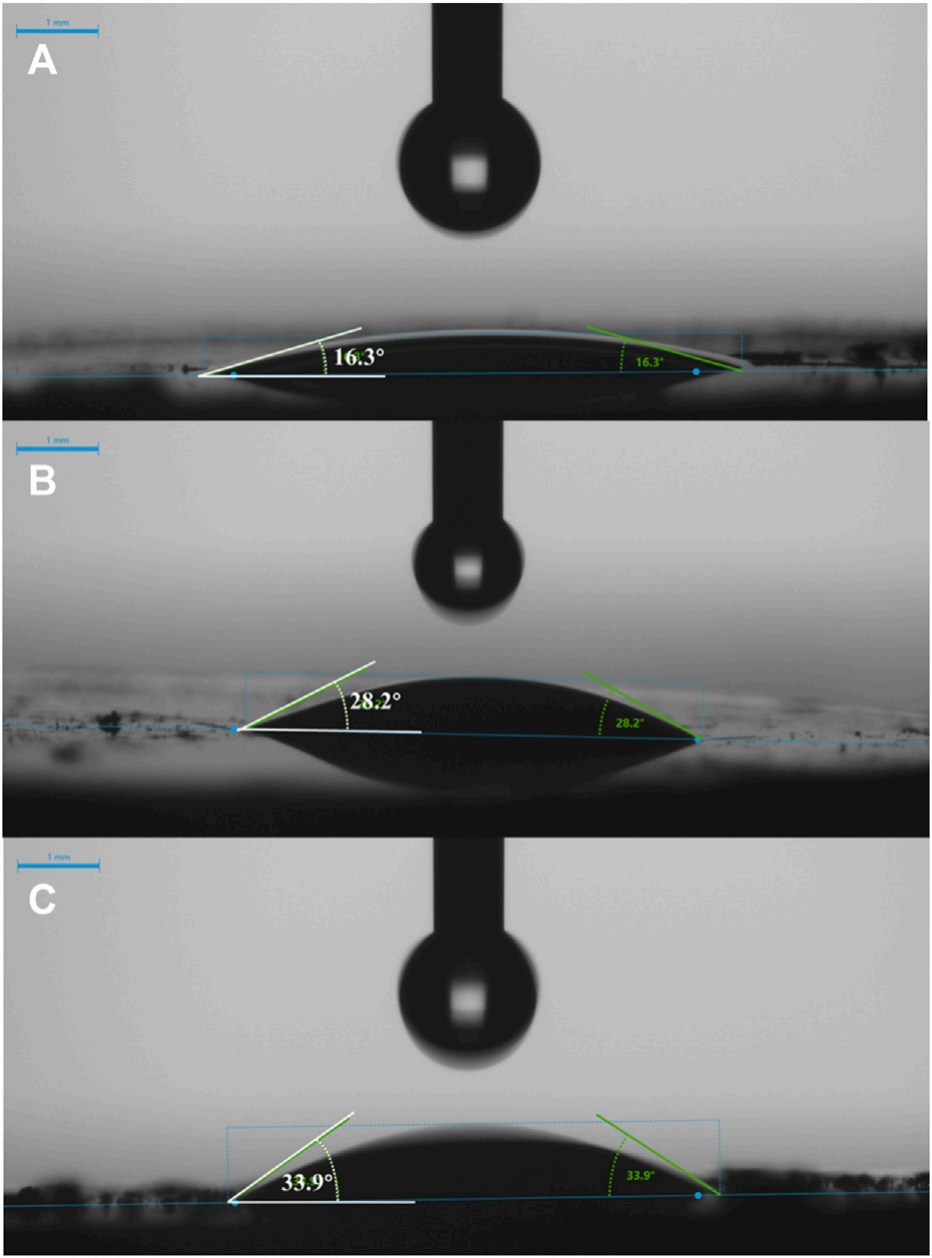
Contact angles of **(A)** ZSM-5 (50), **(B)** ZSM-5 (130), and **(C)** ZSM-5 (360).

In addition, the adsorption in aqueous solution depends on the interactions among zeolite, MTBE, and water molecules, which is closely related to the hydrophobicity of zeolite. As shown in Figure 2, the contact angles of zeolites were in the order of ZSM-5 (360)>ZSM-5 (130)>ZSM-5 (50), indicating that the contact angle increased with the increase of SiO_2_/Al_2_O_3_ ratio. The larger the contact angle, the better the hydrophobicity. The hydrophobic pores separated the organic molecules between the aqueous phase and the adsorbent, creating a favorable environment for the adsorption of organic matter (Erdem-Şenatalar et al., 2004; Abu-Lail et al., 2010). That is, ZSM-5 (360) with a higher SiO_2_/Al_2_O_3_ ratio had stronger hydrophobicity, which was favorable for MTBE adsorption.

**FIGURE 3:**
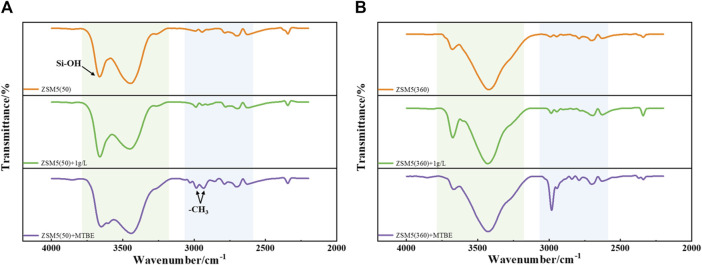
FTIR spectra of **(A)** ZSM-5 (50) and **(B)** ZSM-5 (360) before and after MTBE adsorption.

The infrared spectra of ZSM-5 (50) and ZSM-5 (360) before and after MTBE adsorption are shown in Figure 3. The adsorption peak around 3,700 cm^−1^ is assigned to the silanol group (Sacchetto et al., 2013). The broad band in the range of 3,600–3,200 cm^−1^ can be attributed to the stretching vibrations of H-bonded silanol, which mainly belongs to silanol nests (Braschi et al., 2012). Moreover, new adsorption peaks appeared in the range of 3,100–2,700 cm^−1^ after MTBE adsorption, corresponding to two different methyl groups (methoxy and *tert*-butyl) in MTBE molecules (Martucci et al., 2015), and the signal intensity increased significantly with the increase of MTBE concentration. The other bands with low intensity observed in the range of 3,000–2,400 cm^−1^ can be ascribed to the stretching vibrations of OH groups bridged with H-bond to MTBE molecules. The presence of Brønsted acid sites (Si-OH-Al or HOZ) in ZSM-5 led to the formation of these absorption peaks (Martucci et al., 2015). In summary, silanol was involved in the adsorption of MTBE on ZSM-5, and MTBE was adsorbed on ZSM-5 through H-bond interactions with silanol.

### 3.2 Adsorption mechanism analysis

**FIGURE 4:**
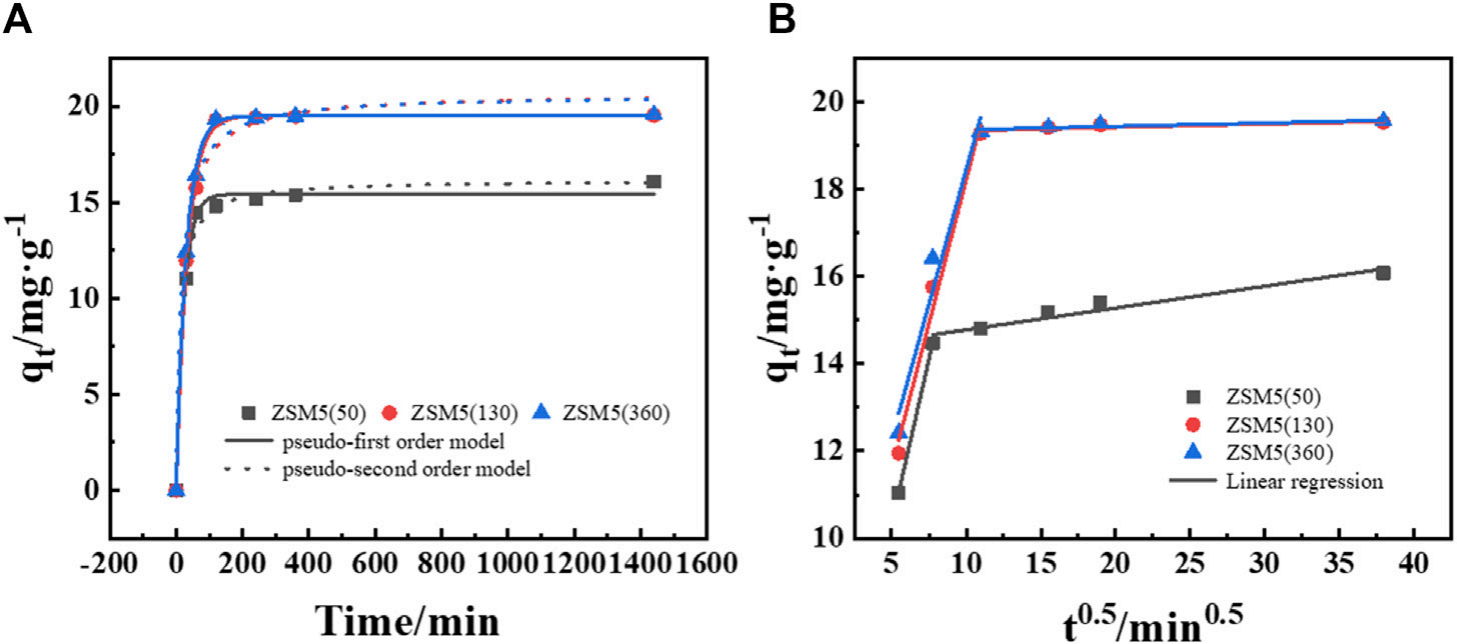
Adsorption process of MTBE on ZSM-5. **(A)** Adsorption kinetics and **(B)** intraparticle diffusion plot at an initial concentration of 100 mg·L^−1^.

The adsorption kinetics of MTBE on ZSM-5 were investigated using pseudo-first and second order models. As shown in Figure 4A, ZSM-5 (50), ZSM-5 (130), and ZSM-5 (360) followed the pseudo-first order kinetic model, indicating that the adsorption rate was proportional to the adsorbate concentration. The adsorption of MTBE on ZSM-5 was rapid at the beginning, reaching about 92%–98% of the equilibrium adsorption capacity within 120 min, and then slowed down with the increase of time. It was found that 1,440 min was sufficient to reach the adsorption equilibrium.

Besides, the data were fitted to the Weber’s diffusion model to understand the mass transfer process (Figure 4B). First, MTBE molecules migrate from the bulk solution through liquid film and are adsorbed on the external surface of ZSM-5. Then, MTBE molecules transport within the pores and are adsorbed onto the internal active sites (Grandjean et al., 2020). The plot of q_t_ versus t^0.5^ represents the different stages of adsorption. If intraparticle diffusion is the only rate-limiting step, the curve will pass through the origin. It was clear that the plot was multilinear and did not pass through the origin, suggesting that both film and intraparticle diffusion were involved in MTBE adsorption (Zhang et al., 2018). The first, sharper region was film diffusion, where MTBE molecules needed to overcome the resistance of the boundary layer. The time for film diffusion was about 60 min for ZSM-5 (50) and about 120 min for ZSM-5 (130) and ZSM-5 (360). Further, the intercept reflects the boundary layer effect. The larger the intercept, the greater the contribution of film diffusion in mass transfer control (Kalavathy et al., 2005; Hameed and El-Khaiary, 2008). As shown in Table 3, the intercepts (c) of ZSM-5 (130) and ZSM-5 (360) were larger, suggesting that film diffusion had greater limitations on the adsorption of MTBE on zeolites with high SiO_2_/Al_2_O_3_ ratios. The second stage was intraparticle diffusion, where MTBE entered the inner pores of ZSM-5 as the external surface became saturated. The reduction in the concentration of MTBE left in solution led to slower intraparticle diffusion. Moreover, the slope of the second linear portion is defined as the intraparticle diffusion parameter K_i_. For ZSM-5 (130) and ZSM-5 (360), the K_i_ values were 0.00088 and 0.00085 mg·g^−1^·min^0.5^ at 50 mg·L^−1^ and 0.0076 and 0.0077 mg·g^−1^·min^0.5^ at 100 mg·L^−1^, respectively. The value of K_i_ increased with the increase of MTBE concentration, indicating that the increase in surface loading improved the driving force of intraparticle diffusion (Hameed and El-Khaiary, 2008). Further, the K_i_ of ZSM-5 (50) was higher, suggesting that the adsorption of MTBE on zeolites with low SiO_2_/Al_2_O_3_ ratios was mainly controlled by intraparticle diffusion.

**TABLE 3:** Kinetic and isothermal parameters of MTBE adsorption on ZSM-5.

Model		ZSM-5 (50)	ZSM-5 (130)	ZSM-5 (360)
Pseudo-first order kinetic model	q_e_/mg·g^−1^	15.43 ± 0.20	19.50 ± 0.17	19.51 ± 0.11
	k/min^−1^	0.0426 ± 0.0032	0.0301 ± 0.0012	0.0327 ± 0.0009
	R^2^	0.9958	0.9983	0.9993
Pseudo-second order kinetic model	q_e_/mg·g^−1^	16.17 ± 0.34	20.69 ± 0.57	20.61 ± 0.52
	k/g·mg^−1^·min^−1^	0.0053 ± 0.0010	0.0025 ± 0.0005	0.0029 ± 0.0005
	R^2^	0.9930	0.9891	0.9904
Intraparticle diffusion model	K_i_/mg·g^−1^·min^0.5^	0.0500 ± 0.0073	0.0076 ± 0.0035	0.0077 ± 0.0024
	C	14.28 ± 0.15	19.27 ± 0.08	19.29 ± 0.06
	R^2^	0.9400	0.7084	0.8336
Langmuir model	q_m_/mg·g^−1^	255.62 ± 19.90	257.86 ± 21.51	270.23 ± 22.60
	b/L·mg^−1^	0.0044 ± 0.0008	0.0068 ± 0.0015	0.0076 ± 0.0017
	R^2^	0.9890	0.9832	0.9831
Freundlich model	K_F_/mg·g^−1^	8.12 ± 3.87	16.46 ± 3.32	18.85 ± 3.54
	1/n	0.4975	0.4065	0.3984
	R^2^	0.9505	0.9890	0.9905

### 3.3 Comparison of MTBE adsorption on ZSM-5 and YK-AC

**FIGURE 5:**
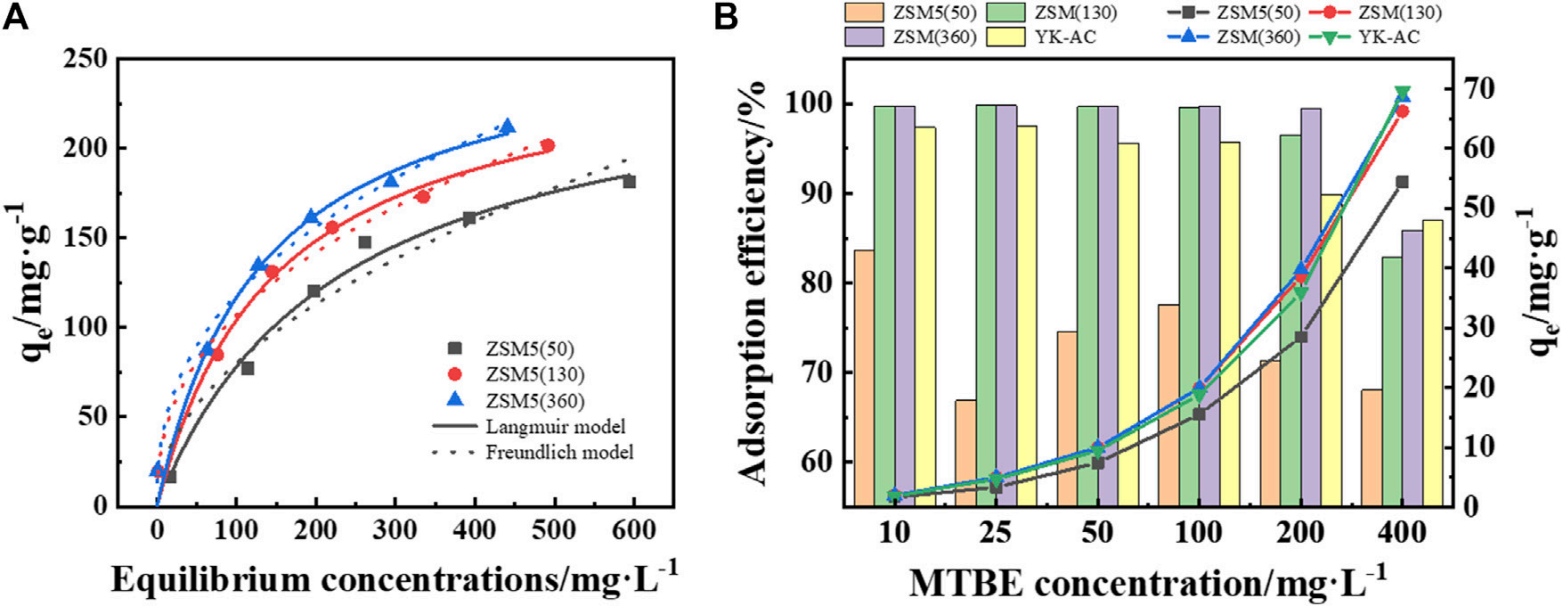
**(A)** Adsorption isotherms of MTBE on ZSM-5. **(B)** Comparison of MTBE removal on ZSM-5 and YK-AC. The dotted line graph represents the adsorption capacity, and the bar graph represents the adsorption efficiency.

The adsorption capacity of ZSM-5 with different SiO_2_/Al_2_O_3_ ratios for MTBE in aqueous solution was investigated using Langmuir and Freundlich isotherms (Figure 5A). As shown in Table 3, the Langmuir isotherm gave the best fit to ZSM-5 (50) with a coefficient R^2^ of 0.9890, indicating monolayer adsorption. The adsorption of MTBE on ZSM-5 (130) and ZSM-5 (360) followed the Freundlich model, indicating inhomogeneous adsorption. The 1/n values were 0.4975, 0.4065, 0.3984 (1 > 1/n > 0), respectively, indicating that the adsorption of MTBE on ZSM-5 was favorable. Moreover, the adsorption capacity increased with the increase of SiO_2_/Al_2_O_3_ ratio, and ZSM-5 (360) achieved the highest adsorption capacity of 270.23 ± 22.60 mg·g^−1^. The results highlighted the influence of SiO_2_/Al_2_O_3_ ratio on MTBE adsorption by ZSM-5. The adsorption of organic pollutants on ZSM-5 was mainly limited by physical properties at high loadings (Anderson Michael, 2000; Erdem-Şenatalar et al., 2004). The larger BET surface area and pore volume facilitated the adsorption of MTBE at high loadings, resulting in the highest maximum adsorption capacity of ZSM-5 (360).

The adsorption efficiency of ZSM-5 and coconut shell-based activated carbon (YK-AC) at lower concentrations was further compared as shown in Figure 5B. The results showed that the adsorption efficiency of ZSM-5 (130) and ZSM-5 (360) for MTBE was higher at initial concentrations less than or equal to 200 mg·L^−1^. There was a substantial difference in adsorption efficiency on ZSM-5 with SiO_2_/Al_2_O_3_ ratios of 50 and 130, whereas the difference between 130 and 360 was less pronounced. Moreover, the adsorption capacity of ZSM-5 (130) and ZSM-5 (360) for MTBE was significantly higher than that of ZSM-5 (50) in the test concentration range (≤400 mg·L^−1^). This was similar to the trend of isotherms, i.e., ZSM-5 with high SiO_2_/Al_2_O_3_ ratios had a higher MTBE adsorption capacity at both low and high concentrations. What’s more, the adsorption efficiency and adsorption capacity of ZSM-5 (360) for MTBE were significantly higher than those of YK-AC at concentrations less than or equal to 200 mg·L^−1^. This suggested that ZSM-5 (360) had the advantage of removing MTBE at low concentrations compared to YK-AC. When the concentration was increased to 400 mg·L^−1^, YK-AC showed the highest adsorption capacity for MTBE, and ZSM-5 (360) was second only to YK-AC. In summary, ZSM-5 (360) showed a strong adsorption capacity for MTBE, especially at low concentrations, and the mechanism was further discussed.

**FIGURE 6:**
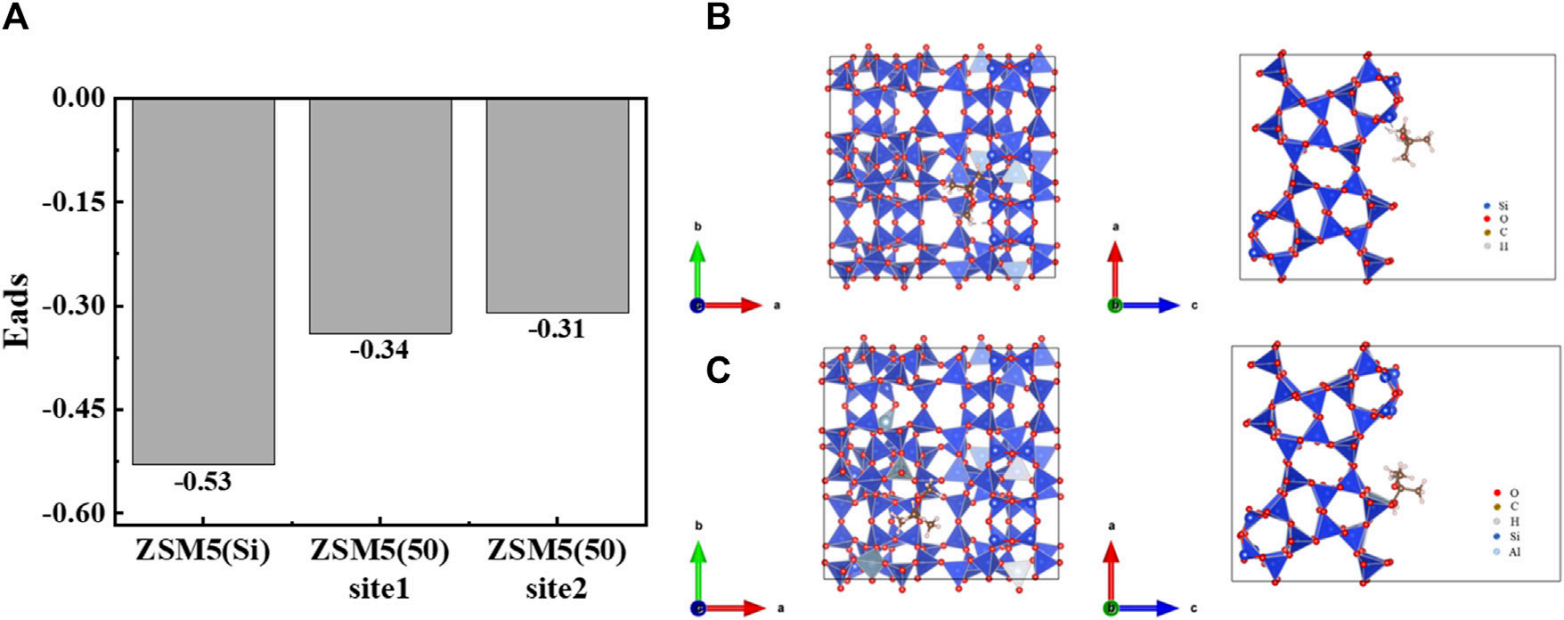
**(A)** Comparison of adsorption energies of different ZSM-5 for MTBE. Structures of **(B)** ZSM-5(Si)-MTBE and **(C)** ZSM-5 (50)-MTBE along *a* and *c* directions, respectively.

In terms of MTBE adsorption on zeolites, the dominant factors favoring adsorption at low concentrations are high SiO_2_/Al_2_O_3_ ratio and high framework density (i.e., hydrophobic pores), whereas large pore volume is more important at high loadings (Arletti et al., 2012; Gonzalez-Olmos et al., 2013). On the one hand, ZSM-5 (360) with a high SiO_2_/Al_2_O_3_ ratio has a hydrophobic surface in the pores. Since the incapability of water to form a condensed liquid phase in the pores, the competition between adsorption of water and MTBE is strongly in favor of organics (Fleys et al., 2004; Zecchina et al., 2007). Furthermore, the strong MTBE-pore wall interaction energies (Erdem-Şenatalar et al., 2004) and the weak tendency of water molecules to interfere with MTBE adsorption in hydrophobic pores result in a high adsorption affinity of ZSM-5 (360) for MTBE (Giaya and Thompson, 2002; Fleys et al., 2004). Besides, according to the DFT calculations (Figure 6A), the adsorption energies were all negative, indicating that MTBE was stably adsorbed on ZSM-5 and the adsorption was favorable (Zhang et al., 2023). Among them, the absolute value of adsorption energy of all-silicon structure ZSM-5 for MTBE was higher, suggesting stronger interaction force and more stable adsorption configuration (Li et al., 2021; Feng et al., 2022). The results confirmed that ZSM-5 (360) had a stronger adsorption capacity among the three zeolites tested.

In addition, the influence of aluminum doping on adsorption was analyzed based on the structure, as shown in Figures 6B, C. The crystal structure of ZSM-5 consists of tetrahedrons of silicon/aluminum and oxygen, forming a silicon/aluminum five-membered ring, and eight symmetric independent five-membered rings form the basic skeleton structure of ZSM-5 (Arletti et al., 2012). The doping of aluminum caused the structure of ZSM-5 to be slightly deformed. On the one hand, the bond length of Al-O bond was elongated and the bond length became different; on the other hand, the bond angle changed, resulting in the five-membered ring no longer being symmetrical. In general, the shorter the bond length, the greater the bond energy, and the stronger the intermolecular interaction force (Cao et al., 2023; Zhou et al., 2023). That is, a stronger interaction force was formed between ZSM-5 with high SiO_2_/Al_2_O_3_ ratios and MTBE molecules, which promoted the adsorption of MTBE. Lu et al. (2008) found that the removal efficiency of all-silica ZSM-5 was higher than that of ZSM-5 (60) at MTBE concentrations ranging from 1,000 to 120 μg·L^−1^. Knappe and Campos (2005) proposed that the SiO_2_/Al_2_O_3_ ratio (90–700) of zeolite had little effect on MTBE adsorption. Li et al. (2023) found that as the SiO_2_/Al_2_O_3_ ratio increased, the adsorption energy increased, and the interaction force between iodine and ZSM-5 was enhanced, promoting the adsorption of iodine. Feng et al. (2024) found that as the SiO_2_/Al_2_O_3_ ratio decreased, compensating cations were introduced, resulting in partial channel occupation of ZSM-5. The introduction of excessive cations could even cause steric hindrance effect, which reduced the adsorption performance of zeolite on large molecular hydrocarbons. Although Al-substituted zeolites had a negative effect on the adsorption of hydrocarbons, the adsorption of H_2_O, CO_2_ and C_2_H_4_O was enhanced. The different results can be attributed to the different properties of the adsorbate molecules. In this study, the differences in MTBE adsorption on ZSM-5 with SiO_2_/Al_2_O_3_ ratios of 50 and 130 at low concentrations (≤200 mg·L^−1^) were significant, while the differences between 130 and 360 was less pronounced. The maximum adsorption capacity of ZSM-5 for MTBE at high loadings increased with the increase of SiO_2_/Al_2_O_3_ ratio. In summary, the adsorption of MTBE on ZSM-5 varied considerably between SiO_2_/Al_2_O_3_ ratios of 0 and 130, with little difference above 130.

### 3.4 Effect of BTEX on MTBE adsorption by ZSM-5 and YK-AC

**FIGURE 7:**
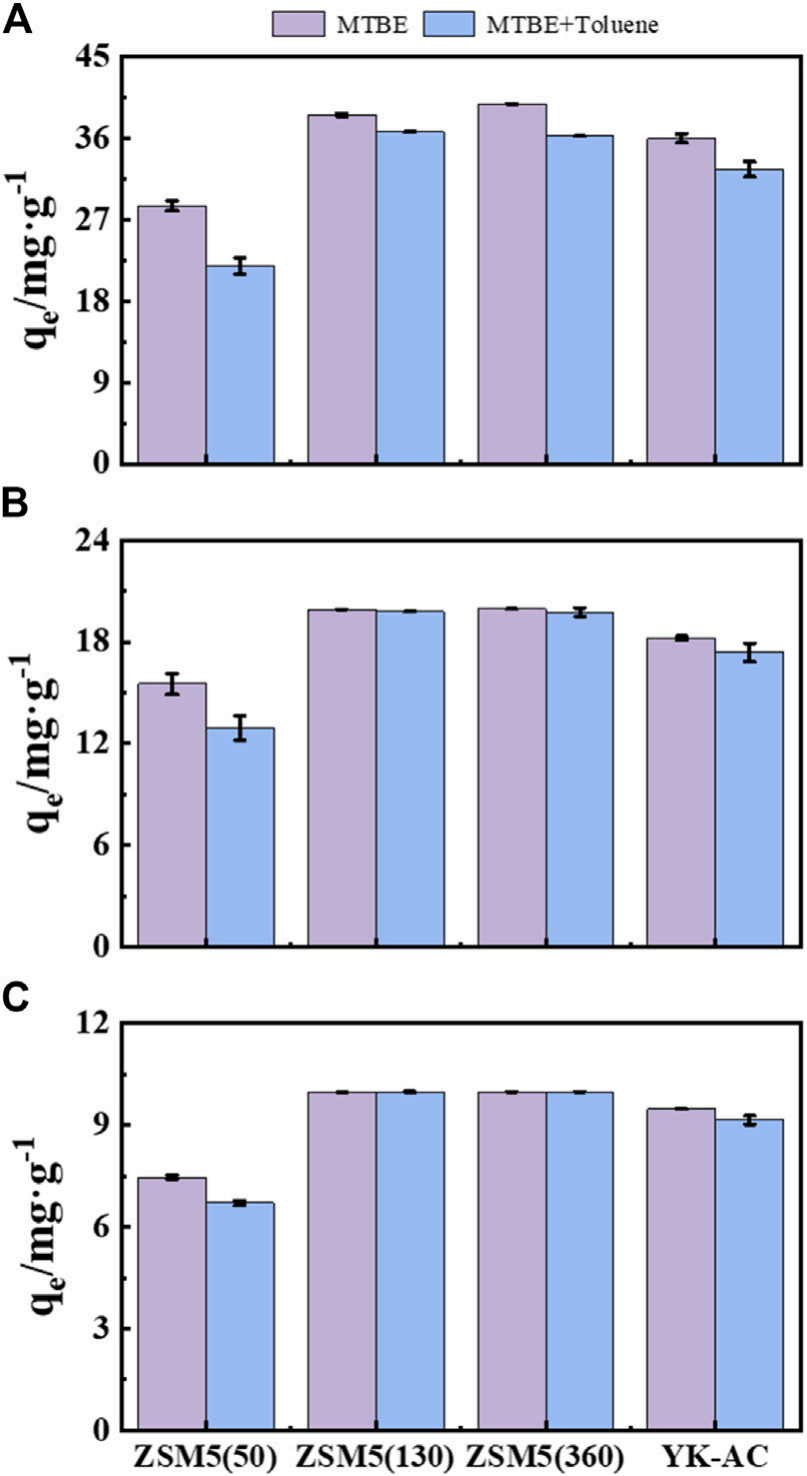
Effect of BTEX on MTBE adsorption by ZSM-5 and YK-AC in mixed contamination. Total concentrations were **(A)** 100, **(B)** 200, and **(C)** 400 mg·L^−1^, respectively.

Competitive adsorption of MTBE with BTEX is more important than single-solute adsorption in water treatment, because MTBE is often accompanied by BTEX in gasoline-contaminated environments (Aivalioti et al., 2012). MTBE and toluene were mixed to different concentrations to compare the adsorption performance of ZSM-5 and YK-AC for MTBE, as shown in Figure 7.

The adsorption capacity of ZSM-5 (50) and YK-AC for MTBE reduced significantly in the mixture of MTBE and toluene at a total concentration up to 200 mg·L^−1^. The adsorption capacity of ZSM-5 (130) and ZSM-5 (360) for MTBE was higher and similar to that without toluene. It indicated that the addition of toluene at low concentrations (≤200 mg·L^−1^) had no significant effect on the adsorption of MTBE on zeolite with high SiO_2_/Al_2_O_3_ ratios. Moreover, the adsorption capacity of three zeolites and YK-AC for MTBE decreased to different degrees at a total concentration of 400 mg·L^−1^. In comparison, ZSM-5 (360) showed the highest removal of MTBE, suggesting that zeolites with high SiO_2_/Al_2_O_3_ ratios had better selective adsorption capacity for MTBE.

The addition of toluene reduced the adsorption capacity of YK-AC for MTBE, indicating that the adsorption of MTBE on YK-AC was inhibited by co-existing organic compounds, which was in agreement with other studies (Hung et al., 2005; Jahandar Lashaki et al., 2023). The physical properties of MTBE (e.g., high water solubility, low K_ow_ value, etc.) resulted in a lower affinity for YK-AC than BTEX. BTEX could compete with MTBE for available adsorption sites or block pores, thus hindering MTBE adsorption on YK-AC (Shih et al., 2003). The better selective adsorption of MTBE on ZSM-5 (360) may be related to the pore structure. ZSM-5 has a 10-membered ring channel structure (minor and major axis dimensions of 5.1 Å × 5.5 Å and 5.4 Å × 5.6 Å for the sinusoidal and straight channels, respectively) (Gonzalez-Olmos et al., 2013). The researchers estimated the dimensions of MTBE to be 5.75 Å × 5.93 Å × 7.2 Å based on a multi-step potential model and concluded that MTBE could be slightly deformed to fit into the pores of ZSM-5 zeolites. The combination of a sufficient number of defects in the crystalline structure and natural vibrations in the lattice facilitated the penetration of MTBE molecules into the pores (Erdem-Şenatalar et al., 2004). Knappe and Campos (2005) showed that the pore structure of ZSM-5 zeolite effectively adsorbed MTBE and had a higher MTBE adsorption capacity than Beta zeolite with larger pores. Due to the molecular characteristics of MTBE and the micropore filling effect, ZSM-5 pores had a stronger adsorption affinity for MTBE (Liu et al., 2021). However, toluene (with an effective diameter of about 6.8 Å, larger than MTBE (Li et al., 2003)) was less compatible with ZSM-5 and could not enter the ZSM-5 channel smoothly (Gonzalez-Olmos et al., 2013). Therefore, zeolites with high SiO_2_/Al_2_O_3_ ratios could effectively adsorb MTBE in co-contamination and had higher adsorption capacity than YK-AC. Similarly, ZSM-5 had a higher adsorption selectivity for aliphatic than aromatic compounds in binary mixtures. The refined host-guest interactions revealed a stronger affinity of ZSM-5 for hexane molecules relative to toluene (Rodeghero et al., 2020). Abu-Lail et al. (2010) showed that the adsorption isotherms of MTBE on ZSM-5 were identical in pure water and NOM-containing conditions, suggesting that ZSM-5 prevented NOM from interfering with MTBE adsorption. Gonzalez-Olmos et al. (2009) found that humic acid (100 mg·L^−1^) had no significant effect on the performance of iron-containing zeolite (Fe-ZSM-5) for the catalytic degradation of MTBE. BTEX, NOM and humic acid were excluded from ZSM-5 pores due to their larger molecular size and poor compatibility with ZSM-5 pores. It was hypothesized that similar macromolecular organics would not compete with MTBE for the adsorption site of ZSM-5, and had little effect on MTBE adsorption. In summary, ZSM-5 (360) had the advantage of selective adsorption of MTBE and high adsorption efficiency at low concentrations due to its skeleton structure, physical properties, and hydrophobicity.

### 3.5 Fixed-bed adsorption of MTBE on ZSM-5 and YK-AC

**FIGURE 8:**
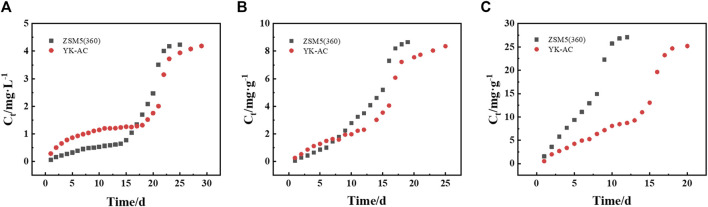
Breakthrough curves of MTBE on ZSM-5 and YK-AC at **(A)** 5, **(B)** 10, and **(C)** 30 mg·L^-1^.

Two adsorbents (YK-AC and ZSM-5 (360)) were loaded onto the column to compare the dynamic adsorption behavior for MTBE. As shown in Figure 8, the MTBE concentration in the effluent of ZSM-5 (360) column was lower during the first 16 and 7 d at concentrations of 5 and 10 mg·L^−1^, respectively. However, as the concentration increased to 30 mg·L^−1^, the effluent concentration of ZSM-5 (360) column increased rapidly and was penetrated earlier. This suggested that ZSM-5 (360) column was suitable for MTBE removal at low concentrations (≤10 mg·L^−1^). Moreover, although the columns were filled with different adsorbents, the breakthrough curves showed the same trend, i.e., the curves shifted towards the origin and became steeper as the initial concentration increased.

Four models were applied to fit breakthrough curves. As shown in Supplementary Table S1, the breakthrough curves followed the Adams-Bohart (A-B), Thomas and Yoon-Nelson (Y-N) models. As the initial concentration increased, the adsorption kinetic constant (k) and the volume of treated water (b) decreased, and the saturation concentration (N) and column adsorption capacity (q) increased. In the A-B model, the adsorption rate of ZSM-5 (360) column for MTBE was always higher, which was more than twice as high as that of YK-AC at 5 mg·L^-1^. The film diffusion rate of ZSM-5 (360) was significantly higher than that of YK-AC at the initial stage, which resulted in a faster mass transfer of ZSM-5 (360) in column adsorption. In the Thomas and D-R models, the column adsorption capacity and treated water volume of the two columns were similar at 5 mg·L^−1^ and higher for YK-AC column at 10–30 mg·L^−1^. Considering the low concentration of MTBE contamination in the environment, the adsorption behavior of the two columns for MTBE at 5 mg·L^−1^ was compared. It was supposed that the treatment goal was to limit the effluent concentration to 0.05% of the influent. According to the Thomas model, ZSM-5 (360) column would be able to treat approximately 13.07 L compared to 0.61 L solution for YK-AC at 5 mg·L^−1^. Obviously, ZSM-5 (360) column achieved better effluent quality with a larger solution volume that met the pollutant limit concentrations. Therefore, it is beneficial to further explore the removal of MTBE on ZSM-5 (360).

The column adsorption parameters were further calculated based on the fitting results of the Y-N model, as shown in Table 4. For YK-AC column, as the concentration increased from 5 to 30 mg·L^−1^, the breakthrough time (t_b_) increased and then decreased, the saturation time (t_s_) shortened by about 34%, the column separation capacity (q_e_) increased by about 3.2-fold, and the MTBE removal efficiency (R) decreased and then increased. As the concentration increased from 5 to 10 mg·L^−1^, the increase in concentration gradient improved the driving force and mass transfer from solution to adsorbent, thus prolonging the breakthrough time. As the concentration increased to 30 mg·L^−1^, the high solute mass per unit area of the adsorbent led to a rapid movement of the adsorption zone, and therefore the breakthrough time decreased (Radhika et al., 2018; Xiang et al., 2023). Due to the higher maximum adsorption capacity of YK-AC for MTBE, it took longer to reach adsorption saturation, had a higher column separation capacity, and was more efficient in removing MTBE at high concentration (30 mg·L^−1^). For ZSM-5 (360) column, as the concentration increased from 5 to 30 mg·L^−1^, the breakthrough time and saturation time reduced by about 96.82% and 54.85%, respectively, and the column separation capacity increased by about 1.2-fold, while the removal efficiency decreased. At low concentrations (5–10 mg·L^−1^), due to the stronger adsorption capacity of ZSM-5 (360) for MTBE, the effluent concentration was lower, and the breakthrough time was significantly prolonged with higher adsorption efficiency. Specifically, the breakthrough time of ZSM-5 (360) column was about 10 times longer than that of YK-AC at 5 mg·L^−1^, suggesting that ZSM-5 (360) maintained a lower effluent concentration over a larger volume of treated solution.

**TABLE 4:** Column adsorption parameters of MTBE on ZSM-5 and YK-AC.

	YK-AC	ZSM-5 (360)	YK-AC	ZSM-5 (360)	YK-AC	ZSM-5 (360)
	5 mg/L		10 mg/L		30 mg/L	
t_b_/h^a^	24.49	262.97	66.39	97.48	61.44	8.35
t_s_/h^b^	773.14	583.82	576.87	459.73	510.72	263.57
m_adsorb_/mg^c^	115.67	114.00	188.062	156.72	488.87	248.31
m_total_/mg^d^	174	150	300	228	720	432
q_e_/mg·g^-1^ ^e^	33.05	32.57	53.73	44.78	139.68	70.95
R/%^f^	66.48	76.00	62.69	68.74	67.90	57.48
LUB/cm^g^	8.71	4.95	7.96	7.09	7.92	8.71
Z/%^h^	3.17	45.04	11.51	21.20	12.03	3.17

^a^ The breakthrough time (t_b_) is established when the effluent MTBE, concentration reaches 5% of the influent concentration (C/C_0_ = 0.05).

^b^ The saturation time (t_s_) was established when the effluent MTBE, concentration exceeded 85% of influent concentration (C/C_0_ = 0.85).

^c^ m_adsorb_: the adsorbent amount of MTBE in the column: $m_{\text{adsorb}}=\frac{Q}{1000}\int_{t=0}^{t=t_{\text{total}}}\left(C_{0}‐C\right)\text{ dt}$

^d^ m_total_: the total amount of MTBE through the column: $m_{\text{total}}=\frac{C_{0}Qt_{\text{total}}}{1000}$

^e^ q_e_: the equilibrium MTBE uptake, also called column separation capacity: $q_{e}=\frac{m_{\text{adsorb}}}{m_{\text{AC}}}$

^f^ R: the total MTBE removal percentage: $R=\frac{m_{\text{adsorb}}}{m_{\text{total}}}\times 100\%$

^g^ LUB: the length of the unused bed: $\text{LUB}=\left(1‐\frac{t_{b}}{t_{s}}\right)L$

^h^ Z: the bed utilization: $Z=\left(1‐\frac{\text{LUB}}{L}\right)\times 100\%$

Besides, it is important to minimize the length of unused bed (LUB) in designing and optimizing fixed-bed columns (Xiang et al., 2023). With the increase of initial concentration, the LUB value of YK-AC column decreased and the bed utilization (Z) increased. On the contrary, the LUB value of ZSM-5 (360) column increased and the bed utilization decreased. Obviously, the application conditions of ZSM-5 (360) and YK-AC were different. For ZSM-5 (360) column, the LUB values were 4.95, 7.09, 8.71 cm, and the bed utilization were 45.04%, 21.20% and 3.17%, respectively, at initial concentrations of 5, 10 and 30 mg·L^−1^. Importantly, the bed utilization of ZSM-5 (360) column was higher at low concentrations, with an improvement of about 42% at 5 mg·L^−1^ and about 10% at 10 mg·L^−1^ compared to YK-AC. This indicated that ZSM-5 (360) column improved the bed utilization at 5–10 mg·L^−1^, which could reduce the energy cost in the dynamic adsorption process, while elevated concentration was more favorable for the adsorption of MTBE on YK-AC. In summary, ZSM-5 (360) column efficiently removed MTBE at low concentrations, with lower effluent concentration and higher removal efficiency and bed utilization. Considering the low concentration of MTBE contamination in the environment, ZSM-5 (360) has a greater potential for practical application. In addition to raw materials, zeolite composites have received a lot of attention. Different zeolite composites were prepared using oil palm ash synthesized with AC (Khanday et al., 2017b), electric arc furnace steel slag (Khanday and Hameed, 2018), and chitosan (Khanday et al., 2017a), respectively, all of which were effective in removing contaminants from aqueous solutions. That is, it is promising to synthesize ZSM-5 composites with high BET surface area to extend the adsorption advantages of ZSM-5 (360) for MTBE.

## 4 Conclusion

In this study, ZSM-5 with SiO_2_/Al_2_O_3_ ratios of 50, 130, and 360 were selected for characterization and MTBE adsorption. The SiO_2_/Al_2_O_3_ ratio affected the mass transfer of MTBE on ZSM-5. The adsorption of MTBE on ZSM-5 with low SiO_2_/Al_2_O_3_ ratios was monolayer, which was greatly influenced by intraparticle diffusion. The adsorption of MTBE on ZSM-5 with high SiO_2_/Al_2_O_3_ ratios was heterogeneous and mainly limited by film diffusion. Moreover, the SiO_2_/Al_2_O_3_ ratio changed the physicochemical properties and skeleton structure of ZSM-5. The larger BET surface area, pore volume, and stronger hydrophobicity of ZSM-5 with high SiO_2_/Al_2_O_3_ ratios enhanced the adsorption of MTBE. Aluminum doping caused slight deformation of ZSM-5 structure, which weakened the interaction force with MTBE molecules and was not conducive to the adsorption of MTBE. Compared with YK-AC, ZSM-5 (360) showed higher adsorption efficiency for MTBE at low concentrations, and had the advantage of selective adsorption of MTBE. Besides, the column packed with YK-AC enhanced the adsorption of MTBE at elevated concentrations, with a column separation capacity of 139.68 mg·g^−1^. ZSM-5 (360) column was more effective in removing MTBE at low concentrations (≤10 mg·L^−1^), with lower effluent concentration and higher removal efficiency, increasing bed utilization by about 42% at 5 mg·L^−1^. In conclusion, ZSM-5 with high SiO_2_/Al_2_O_3_ ratios (≥130) had advantages in removing MTBE in low concentration and mixed contamination, and has greater practical application potential.

## Data Availability

The raw data supporting the conclusions of this article will be made available by the authors, without undue reservation.
