# Supplementary material for: Effective and selective adsorption of methyl tert-butyl ether on ZSM-5 zeolite: a comparative study
Source: Front Chem. 2024 Aug 16;12:1450233. doi: 10.3389/fchem.2024.1450233 (PMC11362031; doi:10.3389/fchem.2024.1450233)
Supplement: Supplementary file 1 [file DataSheet1.docx]

Supplementary Material

# Supplementary Methods

Adams-Bohart model was developed based on the assumption that the adsorption rate is proportional to the residual capacity of adsorbent and the concentration of adsorbate (Goel et al., 2005; Zhang et al., 2019). The expression can be written down as follows:

$\frac{\text{C}_{\text{t}}}{\text{C}_{\text{0}}}\text{=}\frac{\text{e}^{\text{k}\text{C}_{\text{0}}\text{t}}}{\text{e}^{\text{(kN}\text{Z}/\text{v}\text{)}}\text{-1+}\text{e}^{\text{k}\text{C}_{\text{0}}\text{t}}}$ (1)

where C_t_ is the MTBE concentration in the effluent at time t (mg∙L^−1^), C_0_ is the inlet MTBE concentration (mg∙L^−1^), k is the adsorption rate constant (L∙mg^−1^∙h^−1^), v is the linear flow rate (cm∙h^−1^), N is the saturation concentration (mg∙L^−1^) and Z is the bed depth in the column (cm).

Thomas model is commonly used to describe the dynamic behavior of solute adsorption in fixed-bed adsorption systems (Soetaredjo et al., 2014). The suitability of Thomas model can be demonstrated by its assumption regarding negligible axial dispersion in column adsorption. The equation is designated as follows (Thomas, 1944):

$\frac{\text{C}_{\text{t}}}{\text{C}_{\text{0}}}\text{=}\frac{\text{1}}{\text{1+}\text{e}^{\frac{\text{k}_{\text{Th}}}{\text{v}}\text{(qm-}\text{C}_{\text{0}}\text{V)}}}$ (2)

where q is the adsorption capacity (mg∙g^−1^), k_Th_ is the adsorption rate constant (L∙mg^−1^∙min^−1^), v is the flow rate (mL·h^-1^), V is the effluent volume (L), and m is the dosage mass of adsorbent (g).

The widespread use of Yoon and Nelson model in single adsorbate systems is attributed to its simplicity, that is, no detailed data on the adsorbate, adsorbent and the column are required (Yoon and Nelson, 1984). The equation is given by:

$\frac{\text{C}_{\text{t}}}{\text{C}_{\text{0}}}\text{=}\frac{\text{1}}{\text{1+}\text{e}^{\text{k}_{\text{YN}}\text{(T-t)}}}$ (3)

where T is the time required to reach half the adsorbate breakthrough concentration (d) and k_YN_ is the rate constant (min^−1^).

Dose-Response model is an empirical model that has been widely used to describe the kinetics behavior of the column (Tejedor et al., 2023). The general equation is as follows:

$\frac{\text{C}_{\text{t}}}{\text{C}_{\text{0}}}\text{=1-}\frac{\text{1}}{\text{1+}{\text{(}\frac{\text{C}_{\text{0}}\text{V}}{\text{qm}}\text{)}}^{\text{a}}}$ (4)

where a is the constant, V is the effluent volume (L), and q is the amount of MTBE adsorbed onto adsorbent at equilibrium (mg·g^-1^).

# Supplementary Figures and Tables

**Table S1.** Model fitting parameters for MTBE dynamic adsorption on ZSM-5(360) and YK-AC.

| Model | | YK-AC | ZSM-5(360) | YK-AC | ZSM-5(360) | YK-AC | ZSM-5(360) |
| --- | --- | --- | --- | --- | --- | --- | --- |
|  |  | 5 mg·L^-1^ | | 10 mg·L^-1^ | | 30 mg·L^-1^ | |
| A-B | k/L·mg^-1^·h^-1^ | (12.18±1.41) ×10^-4^ | (28.94±2.43) ×10^-4^ | (9.18±0.68) ×10^-4^ | (12.88±0.79) ×10^-4^ | (3.54±0.36) ×10^-4^ | (6.06±0.54) ×10^-4^ |
|  | N/mg·L^-1^ | 17813.57 ± 661.44 | 16451.57 ± 207.51 | 27641.64 ± 584.99 | 23122.43 ± 338.17 | 73596.94 ± 1943.77 | 36410.32±1026.17 |
|  | R² | 0.8492 | 0.9600 | 0.9612 | 0.9789 | 0.9315 | 0.9713 |
| Thomas | k/L·mg^-1^·h^-1^ | (12.18±1.41) ×10^-4^ | (28.94±2.43) ×10^-4^ | (9.18±0.68) ×10^-4^ | (12.88±0.79) ×10^-4^ | (3.54±0.36) ×10^-4^ | (6.06±0.54) ×10^-4^ |
|  | q/mg·g^-1^ | 35.40±1.27 | 33.20±0.42 | 55.36±1.18 | 46.51±0.68 | 147.53±3.87 | 72.43±2.12 |
|  | R² | 0.8492 | 0.9600 | 0.9612 | 0.9789 | 0.9315 | 0.9713 |
| Y-N | k/d^-1^ | 0.15±0.02 | 0.35±0.03 | 0.22±0.0163 | 0.31±0.02 | 0.25±0.02 | 0.44±0.04 |
|  | T/d | 20.65±0.74 | 19.37±0.24 | 16.15±0.34 | 13.56±0.20 | 14.34±0.38 | 7.04±0.21 |
|  | R² | 0.8492 | 0.9600 | 0.9612 | 0.9789 | 0.9315 | 0.9713 |
| D-R | b/L | 24.38±1.42 | 23.09±0.34 | 18.83±0.61 | 15.86±0.38 | 16.84±0.68 | 7.99±0.41 |
|  | q/mg·g^-1^ | 34.83±2.03 | 32.99±0.49 | 53.79±1.75 | 45.30±1.08 | 144.31±5.84 | 68.49±3.55 |
|  | R² | 0.7289 | 0.9433 | 0.9225 | 0.9532 | 0.8719 | 0.9263 |

# Reference

Goel, J., Kadirvelu, K., Rajagopal, C., Kumar Garg, V., 2005. Removal of lead(II) by adsorption using treated granular activated carbon: Batch and column studies. J. Hazard. Mater. 125(1), 211-220. <https://doi.org/10.1016/j.jhazmat.2005.05.032>.

Soetaredjo, F.E., Kurniawan, A., Ong, L.K., Widagdyo, D.R., Ismadji, S., 2014. Investigation of the continuous flow sorption of heavy metals in a biomass-packed column: Revisiting the Thomas design model for correlation of binary component systems. RSC Advances. 4(95), 52856-52870. <https://doi.org/10.1039/c4ra06425a>.

Tejedor, J., Álvarez-Briceño, R., Guerrero, V.H., Villamar-Ayala, C.A., 2023. Removal of caffeine using agro-industrial residues in fixed-bed columns: Improving the adsorption capacity and efficiency by selecting adequate physical and operational parameters. J. Water Process Eng. 53, 103778. <https://doi.org/10.1016/j.jwpe.2023.103778>.

Thomas, H.C., 1944. Heterogeneous Ion Exchange in a Flowing System. J. Am. Chem. Soc. 66(10), 1664-1666. <https://doi.org/10.1021/ja01238a017>.

Yoon, Y.H., Nelson, J.H., 1984. Application of Gas Adsorption Kinetics I. A Theoretical Model for Respirator Cartridge Service Life. Am. Ind. Hyg. Assoc. J. 45(8), 517-524.

Zhang, Y., Jin, F., Shen, Z., Wang, F., Lynch, R., Al-Tabbaa, A., 2019. Adsorption of methyl tert-butyl ether (MTBE) onto ZSM-5 zeolite: Fixed-bed column tests, breakthrough curve modelling and regeneration. Chemosphere. 220, 422-431. <https://doi.org/10.1016/j.chemosphere.2018.12.170>.
